# Synthesis and Characterization of Mechanical Properties and Wire Cut EDM Process Parameters Analysis in AZ61 Magnesium Alloy + B_4_C + SiC

**DOI:** 10.3390/ma14133689

**Published:** 2021-07-01

**Authors:** Thanikodi Sathish, Vinayagam Mohanavel, Khalid Ansari, Rathinasamy Saravanan, Alagar Karthick, Asif Afzal, Sagr Alamri, C. Ahamed Saleel

**Affiliations:** 1 Department of Mechanical Engineering, Saveetha School of Engineering, Saveetha Institute of Medical and Technical Sciences (SIMATS), Chennai 602105, India; r.sarravanan@gmail.com; 2 Centre for Materials Engineering and Regenerative Medicine, Bharath Institute of Higher Education and Research, Chennai 600073, India; mohanavel2k16@gmail.com; 3 Department of Civil Engineering, Yeshwantrao Chavan College of Engineering, Nagpur 441110, India; khalidshamim86@rediffmail.com; 4 Department of Electrical and Electronics Engineering, KPR Institute of Engineering and Technology, Coimbatore 641407, India; karthick.power@gmail.com; 5 Department of Mechanical Engineering, P. A. College of Engineering, Visvesvaraya Technological University, Mangaluru 574153, India; 6 Mechanical Engineering Department, College of Engineering, King Khalid University, P.O. Box 394, Abha 61421, Saudi Arabia; salamri@kku.edu.sa (S.A.); ahamedsaleel@gmail.com (C.A.S.); 7 Department of Mechanical Engineering, The University of Akron, Akron, OH 44325-3903, USA

**Keywords:** wire cut electrical discharge machining, tensile strength, wire feed rate, AZ61, surface roughness, material removal rate (MRR)

## Abstract

Wire Cut Electric Discharge Machining (WCEDM) is a novel method for machining different materials with application of electrical energy by the movement of wire electrode. For this work, an AZ61 magnesium alloy with reinforcement of boron carbide and silicon carbide in different percentage levels was used and a plate was formed through stir casting technique. The process parameters of the stir casting process are namely reinforcement %, stirring speed, time of stirring, and process temperature. The specimens were removed from the casted AZ61 magnesium alloy composites through the Wire Cut Electric Discharge Machining (WCEDM) process, the material removal rate and surface roughness vales were carried out creatively. L 16 orthogonal array (OA) was used for this work to find the material removal rate (MRR) and surface roughness. The process parameters of WCEDM are pulse on time (105, 110, 115 and 120 µs), pulse off time (40, 50, 60 and 70 µs), wire feed rate (2, 4, 6 and 8 m/min), and current (3, 6, 9 and 12 Amps). Further, this study aimed to estimate the maximum ultimate tensile strength and micro hardness of the reinforced composites using the Taguchi route.

## 1. Introduction

Manufacturing units need more effective materials to produce novel components for suitable application; this need is satisfied by preparing two or more materials in the composite nature. These composites are mainly used as hopeful materials, the metal matrix composites (MMCs) functionally used to improve the more strength and good wear characteristics. The investigation made on the AZ61 magnesium alloys with reinforced alumina (Al_2_O_3_) by 2 wt% and silicon carbide (SiC) of 0.5, 1, 1.5 wt% nanoparticle hybrid. The excellent hardness, tensile and compressive strength of the composites are concerned with a direct correlation with the reinforcement of SiC filling, the maximum values are found by the addition of 1 wt% of SiC along with AZ61 + 2 wt% Al_2_O_3_ hybrid composite [1].

The Wire Electrical Discharge Machining process of Shape Memory Alloys surface analysis was carried out [2]. For their investigations the results designate that, for the duration of WCEDM (Wire Cut Electrical Discharge Machining) of nitinol Shape Memory Alloys (SMA), proper discharge power is required to accomplish the optimal surface roughness. The compression test considering the material of ZK60 magnesium alloy with different temperature ranges such as 200–400 °C, also considering the strain range of 0.001–1 s^−1^ was conducted [3] For their result, ZK60 magnesium alloy flow stress can be decreased by increasing deformation temperature as well as reduction of strain rate. Using of nano-sized particles can increase the strength of the metal matrix and also sustain the ductility of the aluminum matrix alloy [4]. The authors considered micro level of silicon carbide and nano level of copper oxide with different weight percentages. The base material and reinforced particles were pressed by cold compacting method and finally the green compacts were sintered. They found the excellent tensile strength and micro hardness of the sintered composites; further, they revealed the microstructure by use of scanning electron microscope (SEM).

Magnesium matrix composites with use of silicon carbide reinforced particles are carried out by stir casting process [5]. They used different particles in size levels such as micron level, submicron level and nano level. For their investigation, they chose the stir casting process and extrusion process to improve the magnesium composite strength. They found that the accumulation of different sizes of SiC particles directly improve the ratio of DRXed nucleation. Similarly it can reduce the typical particle size of the matrix. The tensile strength of the composites also improved compared to without addition of SiC material.

The Mechanical Properties are improved in the aluminum alloy with addition of nano B_4_C nano composites by the way of stir casting process [6]. In the microstructure analysis, the boron carbide nano particles disseminated to the aluminum alloy with excellent condition were identified. Increasing of nano boron carbide particles extensively increased the mechanical properties such as tensile strength and hardness. They revealed that the result of dry sliding wear resistance of the nano B_4_C composites was higher than monolithic aluminum alloys.

Machining process of magnesium ZC63 alloy with reinforced silicon carbide by use of wire cut EDM process was conducted [7]. The authors considered different parameters to conduct the wire cut EDM. For their result, increasing of the pulse-on time and wire tension parameters increased the material removal rate. On the contrary, the surface roughness was reduced. Analyzing the machining parameters of wire cut EDM process for SKD11 material is one of the critical one due to its hardness level [8]. Surface roughness was modified by the influence of pulse on time and pulse off time parameters. In the material removal rate (MRR) analysis, the wire speed parameter was highly influenced. MRR was increased by increasing of pulse on time and wire speed. In contrast, the surface roughness was decreased. In both approaches such as BPNN-GA and RSM, the BPNN-GA provides precise output results compared to response surface methodology (RSM) approach. The back propagation neural network combining with genetic algorithm methodology predicted the values in perfect mode.

The liquid phase materials offer good mixing of constituent elements achieved by stir casting process by means of stirring nature. The liquid molten state material is finally casted by the required shape with any size. In the stir casting process, the stirring speed plays a major role to mix the elements or reinforced particles to give excellent mechanical strength after casted components. The stirring time is also important for the particular time taken to distribute particles uniformly [9]. For pouring the liquid into the mold, the pouring rate influences the decision of the quality of the casting and also introduces the possibility to form defects [10,11,12,13].

In the wire cut EDM machining process, thin metallic wire is fed into the specimens constantly which is surrounded by passing of dielectric fluid. The continual feed of the wire is controlled by the guides placed on upper and lower positions to give the uniform speed and tempering nature [14,15]. In the WCEDM, the wires are generally made up of brass materials. The WCEDM is a thermo-electrical approach of cutting the samples at any profiles easily; the erosion action can be completed between samples and electrodes with introduction of an electrical spark [16,17,18,19]. The circulated water improves the feed rate of the wires by removal or washes out the debris from the machining zone.

In the wire cut EDM process, the current passes on the wire on one side and on the other end the specimen carrying the current. The wire moves close to the specimen at the time the pulling of electrical charges is introduced; it causes the melting and vaporization in the specimens to cut any profile [20,21,22,23,24]. The MRR and surface roughness analysis is important in the machining process as the tool performance, machine performance as well as parameter involvement have to be measured. In general, the MRR can be calculated by use of removed material weight such as before machining and after machining the weight of the component has to be estimated [25,26,27,28]. Surface roughness is one of the desirable factors for matting or aligning two surfaces without any trouble in the assembly line in the industries [29].

Surface roughness was measured by applying a surface roughness tester in a short time period; it can be varied by the influence of machine vibration, type of tool and material and coolant supply [30]. The limitations of the stir casting process are the conclusion of reinforcement material melting temperature which is renowned as three times that of base metal. Further, it is reported that the agglomeration of reinforcement particles in the molten material is an extremely crucial aspect in the surface finish and crack propagation. All research work focused on analysis of the mechanical properties, especially the tensile strength and micro hardness. Based on the literature study the AZ61 magnesium alloy is chosen for this work, the selected material is stir casted with reinforced of boron carbide and silicon carbide, finally the small size of specimens is cut by use of wire cut EDM [31]. After the wire cut the material removal rate and surface roughness is analyzed for the effect of wire cut process parameters [32]. Further, the mechanical strength such as tensile strength and micro hardness were tested.

## 2. Materials and Methods

The magnesium alloy AZ61 (Parshwamani Metals, Mumbai, Maharashtra, India) has excellent machinability character, lightweight material and also easy casting for any form. This alloy offered good corrosion resistance in all environmental conditions [33]. The contribution of elements in weight percentage is presented in the Table 1. The highest ultimate strength is offered by the AZ61 magnesium alloy, the remarkable mechanical properties such as hardness, shear strength, compressive strength, bearing strength and impact strength are listed in the Table 2.

## 3. Experimental Procedure

**Figure 1:**
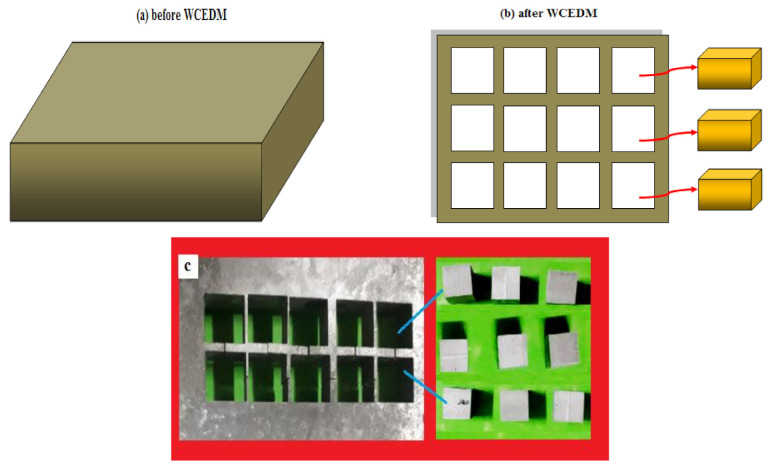
Schematic view of (**a**) casted AZ61Mg specimen (**b**) after WCEDM (Wire Cut Electrical Discharge Machining) specimen and (**c**) casted raw material with machined specimens.

This work was divided into two categories: first the material removal rate and surface roughness analysis was conducted by using L16 orthogonal array (OA) with optimization of wire cut EDM process parameters [34]. Further, the tensile strength and micro hardness were evaluated by optimization of stir casting process parameters. Stir casting process is a novel method to achieve homogeneous mixture of the hybrid composites with effective strength. The hard particles of the boron carbide and silicon carbide particles (Bhukhanvala, Mumbai, Maharashtra, India) were weighed separately, and the reinforcement percentage was considered uniformly such as 8%. AZ61 magnesium alloy of powder form was weighed at the required level in the digital balance (Prolab, Mumbai, Maharashtra, India). Initially the magnesium alloy was melted in the furnace (Vibrant Thermal Engineering, Chennai, Tamil Nadu, India) with elevated temperature, at the time of melting condition the reinforced particles of boron carbide and silicon carbide were put in the furnace and the temperature was maintained around 800 °C. The agitation process enhanced the properties of the hybrid composites as well as increased the strength [35]. The stirring speed of this investigation varied such as 450, 500, 550 and 600 rpm with varying time of stirring such as 15, 20, 25 and 30 min. The pure molten state of the Mg AZ61 was poured in the mold cavity of rectangular shape for the dimension of 200 × 100 × 15 mm^3^, as shown in Figure 1.

The stir casted part was further machined by use of wire cut EDM with selected different cutting factors such as pulse on time (105, 110, 115 and 120 µs), pulse off time (40, 50, 60 and 70 µs), wire feed rate (2, 4, 6 and 8 m/min), and current (3, 6, 9 and 12 Amps). The debris was flushed by use of circulated ionized water into the cutting area improving the cutting action [36,37,38]. Before cutting and after cutting the material was weighed to calculate the MRR of each specimen [39].

### 3.1. Wire Cut EDM Process

The CNC (Computer Numerical Control) Wire EDM Machine (Model: AW6S, Electronica, Kolkata, India) was used to machine the stir casted specimens. Wire tension of the machine was 200–2500 gf, machine operated power supply as 5400 + 350 kg and the movement of the X direction and Y direction distance was 650 and 450 mm, respectively.

Reinforcements of boron carbide and silicon carbide to the AZ61 Mg alloy were highly influenced in the WEDM process. In this investigation it was found that larger reinforced particles considerably reduced the material removal rate, compared to small sized particles it can highly protect the matrix material from EDM sparks. The WEDM process was carried out in the Motto Machining solutions, Coimbatore.

The four process parameters namely pulse on time, pulse off time, wire feed rate and current of the wire cut electric discharge machining process and their chosen values are presented in Table 3. These parameters are highly involved in the wire cut EDM process to improve the MRR and also reduce the surface roughness [40].

### 3.2. Surface Roughness Analysis

**Figure 2:**
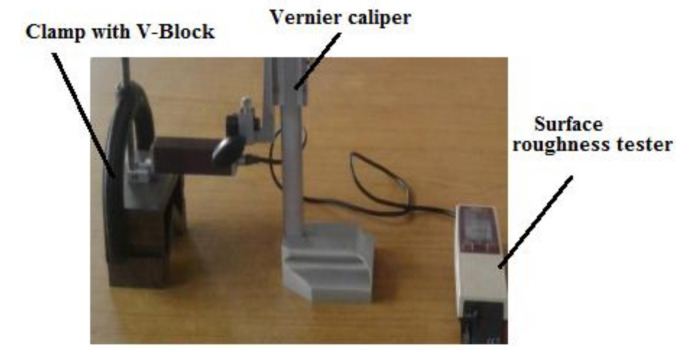
Mitutoyo hardness tester.

The surface roughness was measured by use of Mitutoyo surface roughness testing machine (India Tools and Instruments, Mumbai, India) effectively as shown in Figure 2. Aerospace model Vernier height gauge was used to clamp the surface roughness tester unit; further, the V-block and C-Clamp were used to hold the specimen in the correct position level. All the samples were measured by this instrument to obtain precision results.

### 3.3. Tensile Strength Analysis

Stir casted AZ61 + B_4_C + SiC hybrid composite specimens were tested in the tensile testing machine (Model: ZY 2075–A series, (Blue star Engineering and Electronics, Mumbai, India), maximum of 100 kN capacity and pneumatically operated jaws). The parameters that were selected for the tensile test are reinforcement %, stirring speed, time of stirring, and process temperature as presented in Table 4.

### 3.4. Micro Hardness Analysis

Hardness is one of the major mechanical properties of the materials in engineering application [41]. The micro hardness is the precision measurement of the hardness with the help of Vickers hardness tester (YESAAR TECH, Chennai, India), measuring range: 5–300 HV, for the testing force are 10, 25, 50, 100, 200, 300, 500, 100 gf; magnification of the testing: 500×, 125×; lowest measurement of microscope: 0.025 μm). All the 16 samples were tested with the effects of stir casting parameters.

## 4. Result and Discussion

### 4.1. Material Removal Rate (MRR)

The wire cut EDM was carried out by influence of the elected parameters effectively, the parameters of pulse on time (µs), pulse off time (µs), wire feed rate (m/min) and current (Amps). All the parameters and the output result of material removal rate are summarized and tabulated in Table 5. From the table, maximum material removal rate was obtained as 0.212 mm^3^/s. Table 6 presents the response table for signal to noise ratios of material removal rate. From this table, the wire feed rate parameter has the highest influencing factor in MRR, continually the pulse on time, pulse off time and current. The optimal parameters were recorded as 115 µs of pulse on time, 50 µs of pulse off time, 3 m/min of wire feed rate and 6 Amps of current.

**Figure 3:**
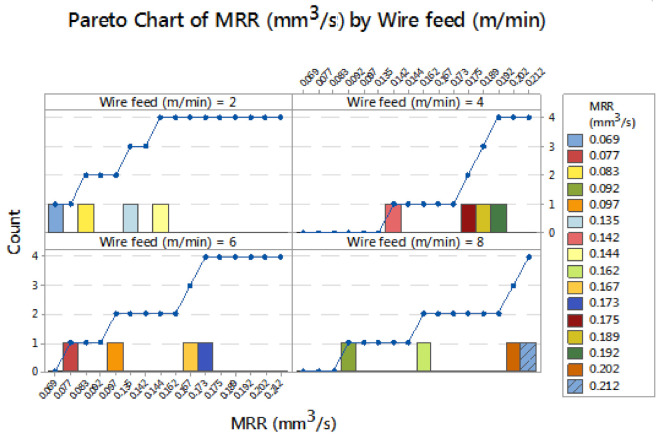
Pareto chart of material removal rate by wire feed rate.

**Figure 4:**
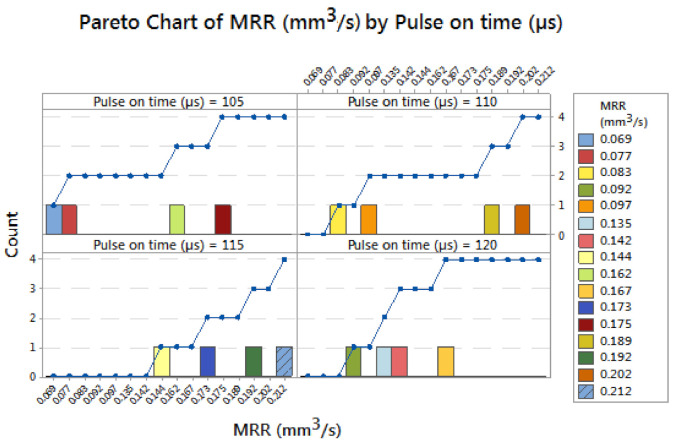
Pareto chart of material removal rate by pulse on time.

In Figure 3, the Pareto chart of material removal rate by wire feed rate is illustrated effectively by the contribution of wire feed rate. The 80/20 rule was implemented for this experiment, influencing the wire feed rate of 2 m/min the minimum MRR was obtained as 0.069 mm^3^/s and 4 m/min of wire feed produced the 0.142 mm^3^/s of MRR. Increasing the wire feed rate of 6 m/min obtained an MRR of 0.077 mm^3^/s. Finally for the 8 m/min wire feed rate the obtained MRR was 0.092 mm^3^/s. All these values are minimum and better for changing the parameters. Figure 4 shows the Pareto chart of material removal rate by influencing pulse on time. Application of minimum pulse on time 105 µs obtained MRRs of 0.069 and 0.077 mm^3^/s. By slightly increasing the pulse on time to 110 µs, the MRR was obtained as 0.083 and 0.097 mm^3^/s. Using 115 µs of pulse on time, the material removal rate was found as 0.142 mm^3^/s. Finally, using 120 µs of pulse on time attained an MRR of 0.092 mm^3^/s. Maximum MRR (0.142 mm^3^/s) can be achieved by using 115 µs of pulse on time.

**Figure 5:**
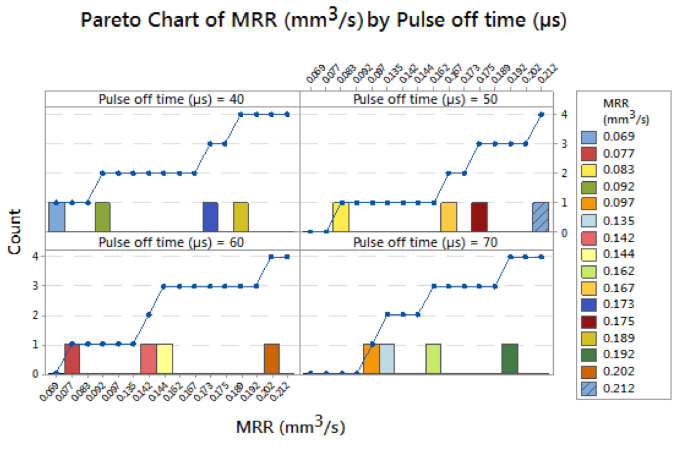
Pareto chart of material removal rate by pulse off time.

**Figure 6:**
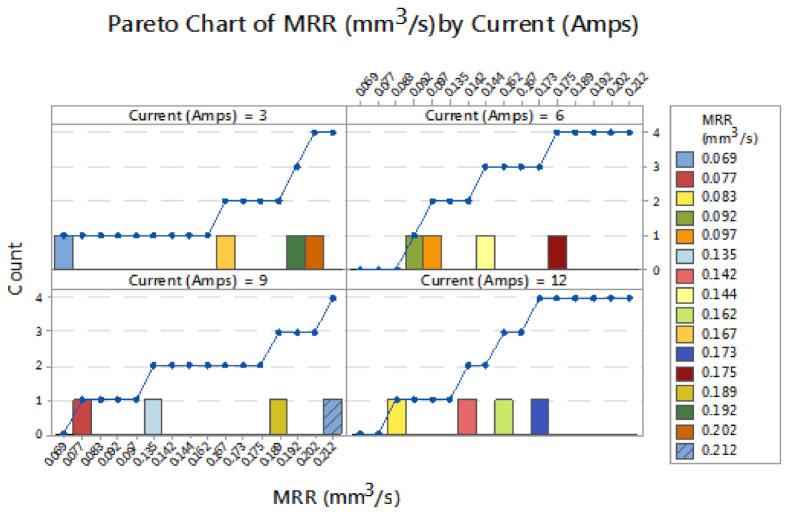
Pareto chart of material removal rate by current (Amps).

Figure 5 illustrates the Pareto chart of material removal rate by pulse off time. Applying pulse off time of 40 µs produced MRRs of 0.069 and 0.092 mm^3^/s. Increasing the pulse off time from 40 to 50 µs acquired an MRR of 0.083 mm^3^/s, continually influencing pulse off time of 60 µs attained an MRR of 0.077 mm^3^/s. Finally, the pulse off time of 70 µs registered an MRR of 0.097 mm^3^/s. Among the four levels of pulse off time, the 70 µs of pulse off time offered the maximum MRR such as 0.097 mm^3^/s. Figure 6 illustrates the Pareto chart of material removal rate by current (Amps) by supplying different current levels such as 3, 6, 9 and 12 Amps. The 3 Amps current recorded a material removal rate of 0.069 mm^3^/s, applying 6 Amps of current obtained an MRR of 0.092 mm^3^/s. Increasing to 9 Amps of current offered 0.077 mm^3^/s of MRR. Finally, the use of 12 Amps current can obtained an MRR of 0.083 mm^3^/s. In all current levels, 6 Amps of current produced the maximum MRR such as 0.092 mm^3^/s. All the minimum values of material removal rate reflected the changing parameters.

**Figure 7:**
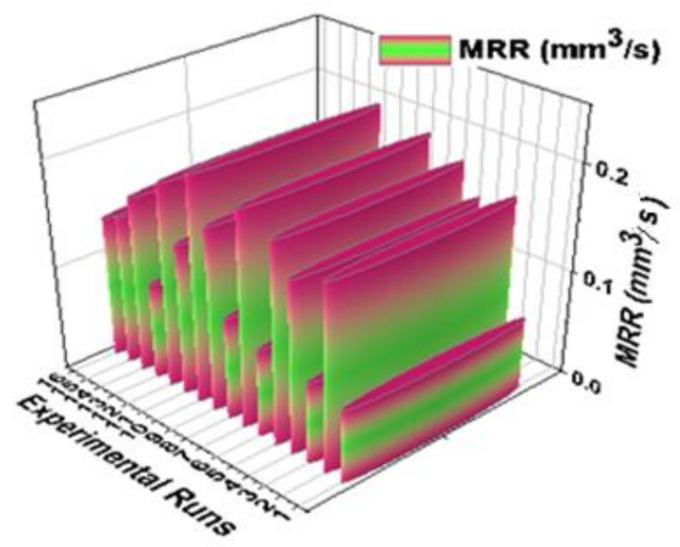
3D bar plot of experimental vs. material removal rate.

Figure 7 illustrates the 3D bar plot of experimental vs. material removal rate effectively. Sixteen experimental runs were carried out and each run presents the minimum and maximum MRR of the AZ61 magnesium alloy hybrid composites. In this analysis, the minimum material removal rate was recorded as 0.069 mm^3^/s by use of 105 µs pulse on time, 40 µs pulse off time, 2 m/min of wire feed rate and 3 Amps of current rate. Similarly, maximum MRR was found to be 0.212 mm^3^/s by use of 115 µs pulse on time, 50 µs pulse off time, 8 m/min of wire feed rate and 9 Amps of current rate. Influencing factors decided the excellent MRR.

**Figure 8:**
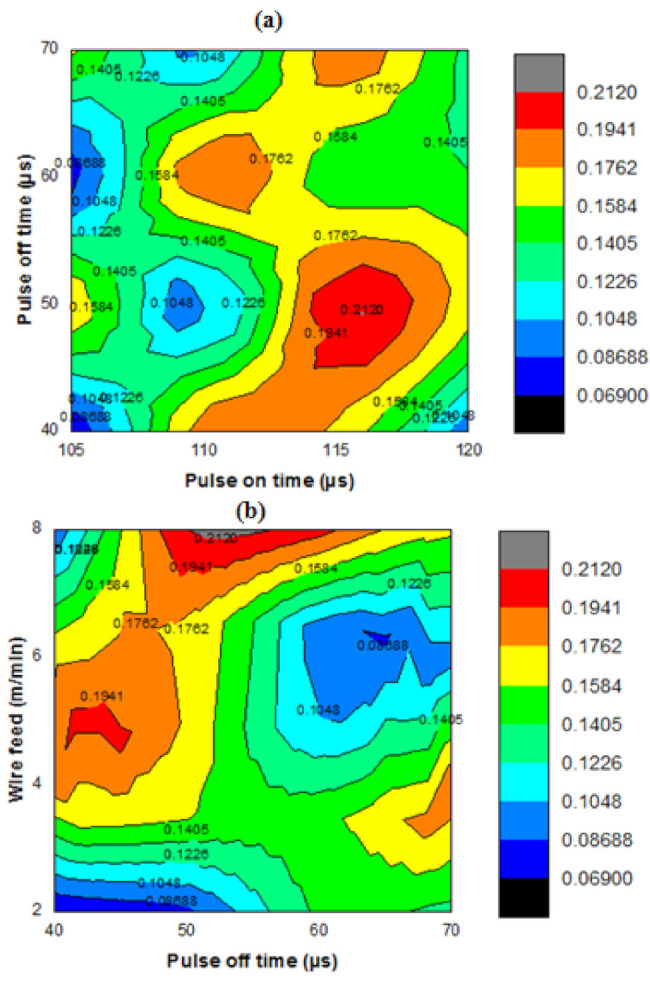

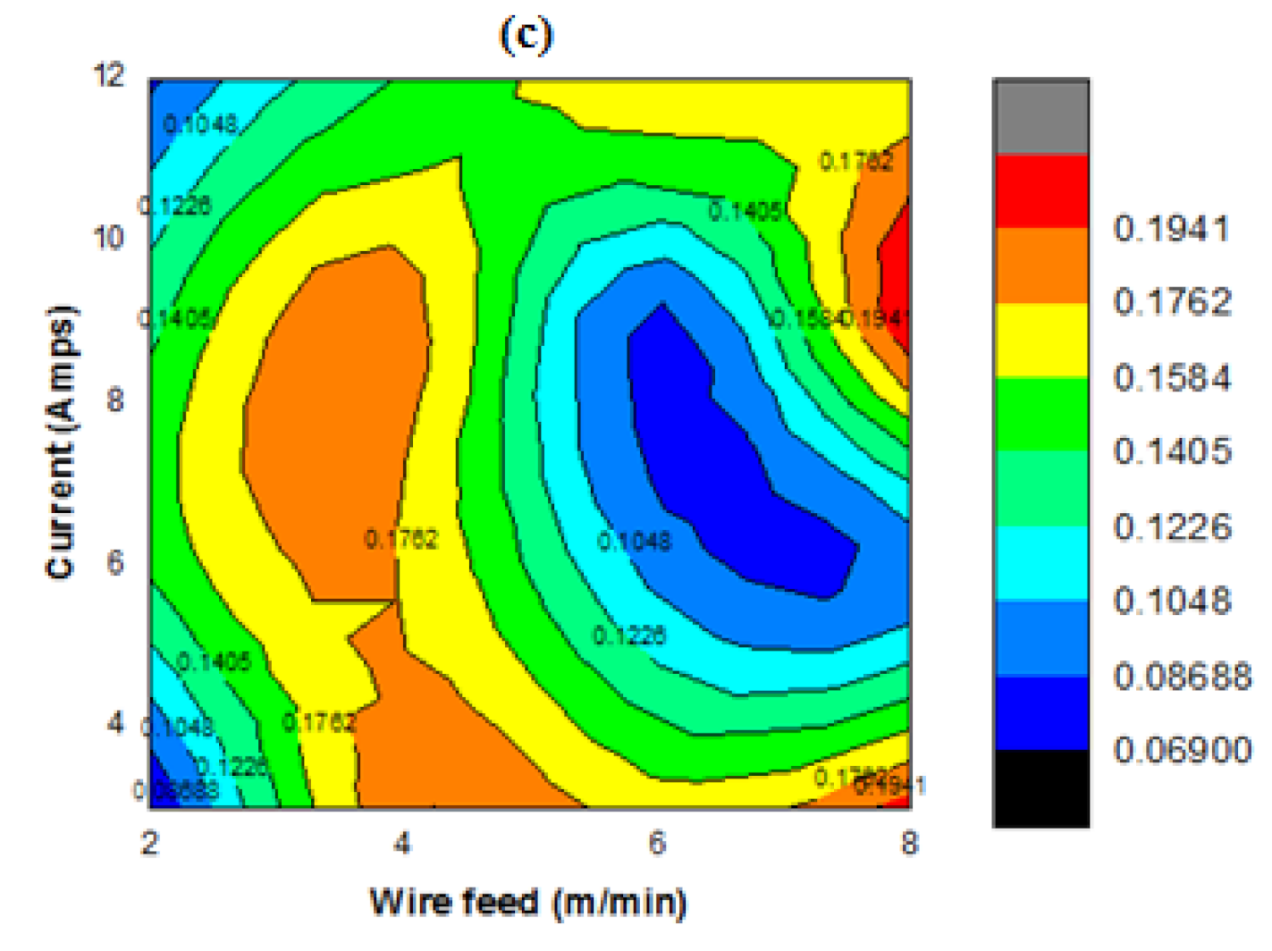
Contour plot of (**a**) pulse on time (µs) vs. pulse off time, (**b**) pulse off time (µs) vs. wire feed rate (m/min) and (**c**) wire feed rate (m/min) vs. current (Amps).

Figure 8a shows the pulse on time (µs) vs. pulse off time (µs) contour plot. The maximum MRR was obtained as 0.2120 mm^3^/s from the influence of the pulse on time of 115 µs and pulse off time of 50 µs. Increasing pulse on time slightly improved the MRR by the way of erosion. Figure 8b shows the contour plot of pulse off time µs vs. wire feed rate m/min. The maximum MRR was obtained with wire feed rate of 8 m/min and the pulse off time of 50 µs. The highest wire feed rate increases the erosion rate, hence MRR was achieved at higher rate such as 0.194 mm^3^/s. Figure 8c shows the wire feed rate (m/min) vs. current (Amps). The highest MRR was acquired at wire feed rate of 8 m/min and the current of 10 Amps. In a similar manner, the wire feed rate was highly influenced as increasing the erosion rate finally obtained maximum MRR.

### 4.2. Surface Roughness Analysis

**Figure 9:**
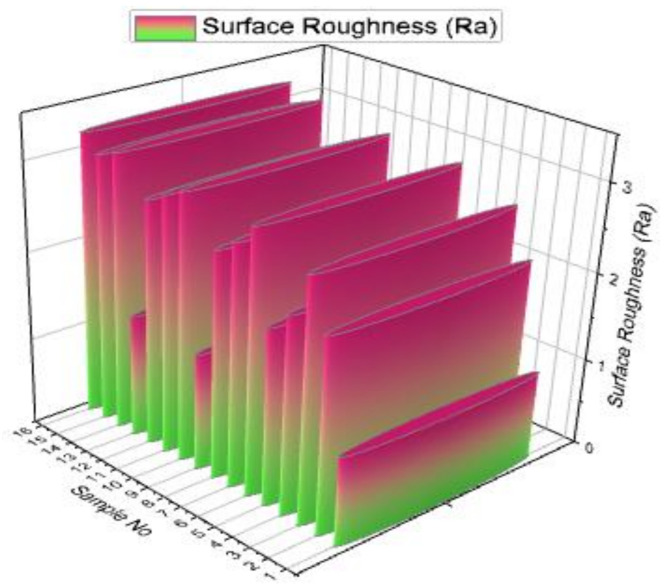
3D bar plot of experimental vs. surface roughness graph.

Figure 9 shows the surface roughness values of the all specimens visibly in the minimum and maximum ranges. Among the 16 specimens, the first specimen has a minimum surface roughness value of 1.0039 microns with influence of pulse on time of 105 µs, pulse off time of 40 µs, wire feed rate of 2 m/min and current of 3 Amps. The specimen number 16 registered a maximum surface roughness value of 3.203 microns. The maximum surface roughness value was attained by the influence of pulse on time (120 µs), pulse off time (70 µs), 2 m/min of wire feed rate and 9 Amps of current. It can be clearly revealed that the increase of pulse on time, pulse off time and the current rate increases the surface roughness. The average surface roughness value was found as 2.442 microns. It can be obtained in the use of L16 Orthogonal Array (OA). From the statistical analysis, the experimental runs 5, 9 and 13 provide the surface roughness values less than 2 microns. On the contrary, the maximum surface roughness above 3 microns was identified in 14, 15 and 16 experimental runs.

Table 7 points out the surface roughness value of the all specimens efficiently. The minimum and maximum values of surface roughness are 1.003942 and 3.203942 microns.

Table 8 illustrates the response table for signal to noise ratios of the surface roughness test. From this table, the pulse off time parameter has the highest influencing factor among all four parameters, further the pulse on time, current and wire feed rate. In surface roughness analysis, the optimal parameters were registered as 105 µs of pulse on time, 40 µs of pulse off time, 8 m/min of wire feed rate and 6 Amps of current.

### 4.3. Tensile Strength Analysis

Table 9 presents the experimental summary of the tensile strength involving the four parameters and four levels; the result of tensile strength was illustrated clearly. The maximum tensile strength was registered as 245 MPa with influencing of reinforcement of 2%, stirring speed of 600 rpm, time of stirring 30 min and processing temperature 800 °C. Increasing stirring time produced the better results.

Table 10 presents the response table for signal to noise ratios of tensile strength. Among four parameters, the stirring speed highly influenced the results in contrast to other parameters. Further, the time of stirring is the second factor of influence in tensile strength, continually followed by processing temperature and reinforcement %.

**Figure 10:**
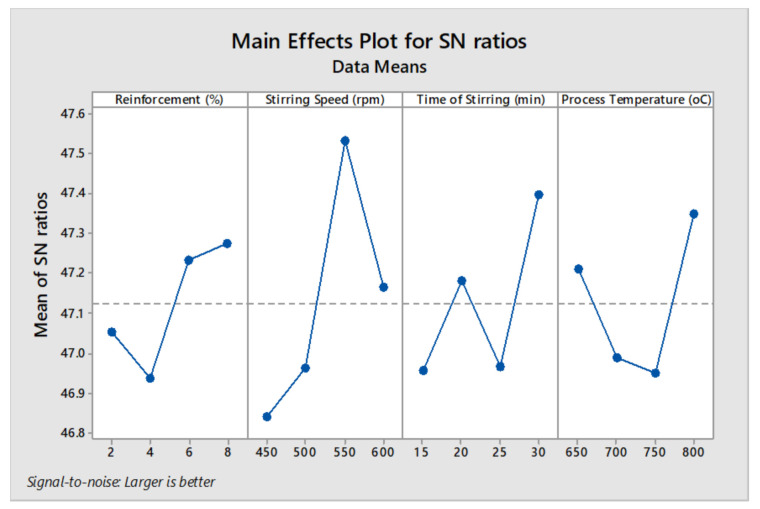
Main effect plot for SN ratios (tensile strength).

Figure 10 shows the main effect plot for SN (signal to noise) ratios of the tensile strength. The increasing reinforcement percentage increased the tensile strength, 8% of reinforcement offered high tensile strength. Among all stirring speed conditions, the third level 550 rpm provided good tensile strength. Minimum tensile strength was viewed in the influence of minimum time of stirring. By increasing the stirring time from 25 to 30 min the higher tensile strengths were obtained [42]. In all processing temperatures, the normal tensile strength was obtained, and 800 °C provided the maximum tensile strength. Optimal parameters were obtained for the tensile strength as 8% of reinforcement, 550 rpm of stirring speed, 30 min of stirring time period and 800 °C of processing temperature.

**Figure 11:**
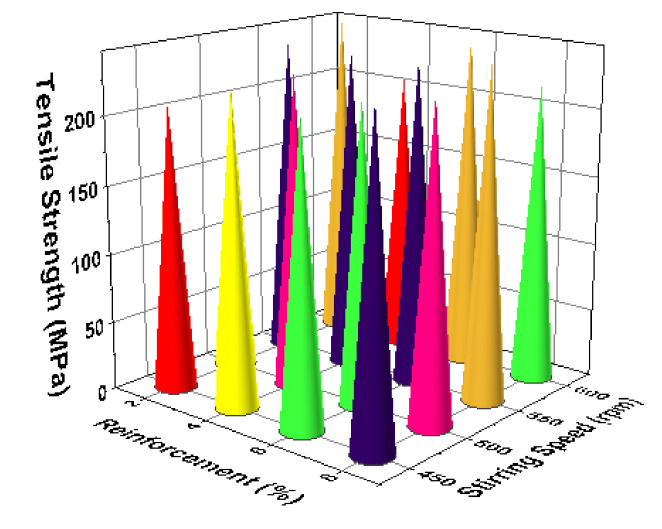
3D bar plot: reinforcement vs. stirring.

**Figure 12:**
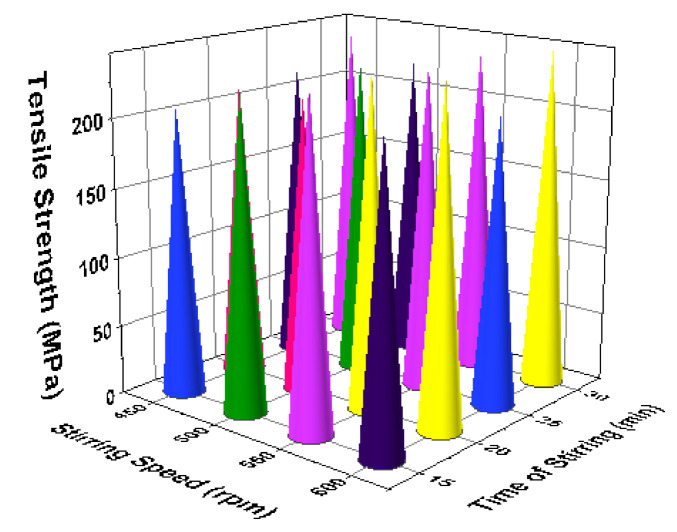
3D bar plot: stirring speed vs. time of stirring.

**Figure 13:**
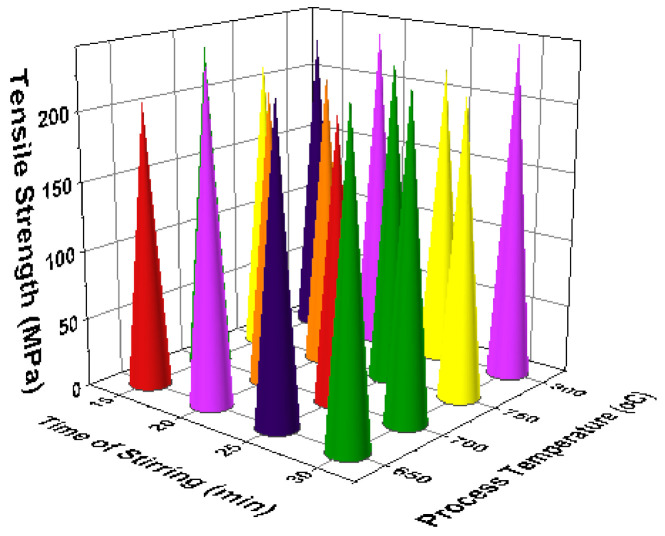
3D bar plot: time of stirring vs. process temperature.

**Figure 14:**
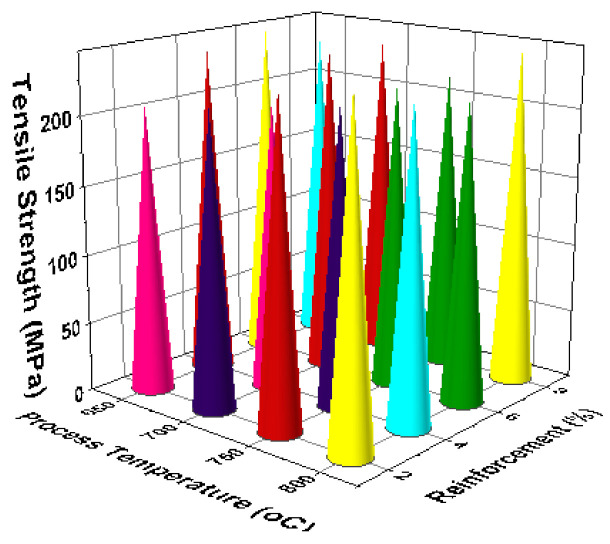
3D bar plot: process temperature vs. reinforcement %.

Figure 11 illustrates the 3D bar plot of tensile strength influenced by two parameters such as reinforcement and stirring speed. This plot shows the increase of stirring speed, as well as increase of reinforcement percentage, highly influenced the increase of tensile strength. Figure 12 presents the two parameters correlation. The minimum stirring speed and the maximum stirring time offered superior tensile strength. Figure 13 presents the correlation between time of stirring and the process temperature. This plot clearly shows that 30 min of time of stirring and 800 °C of processing temperature provided the better tensile strength. Figure 14 illustrates the interaction between two parameters such as process temperature and reinforcement %. The processing temperature of 800 °C and the 8% of reinforcement offered the high tensile strength.

**Figure 15:**
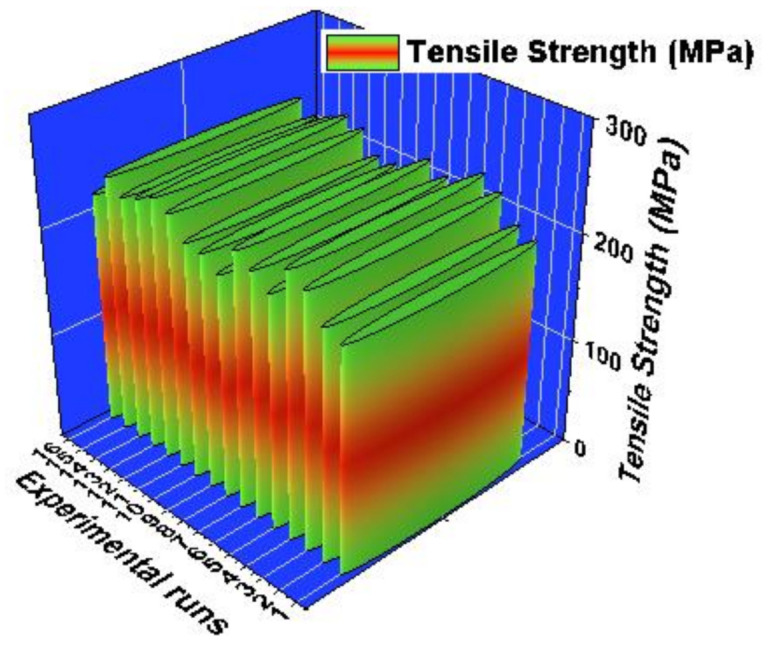
3D bar plot: experimental runs vs. tensile strength.

Figure 15 illustrates the 3D bar plot of experimental vs. tensile strength of the study. In all 16 experimental trials, each trail visibly showed the minimum and maximum tensile strength of the AZ61 hybrid composites. In most of the experimental runs, the tensile strength was nearer to others. For this examination the maximum tensile strength was recorded as 245 MPa. On the contrary, the minimum tensile strength was achieved as 208 MPa. From this analysis the multiple runs offered excellent tensile strength. Stirring speed highly influenced modification of the tensile strength.

### 4.4. Micro Hardness Analysis

Table 11 presents the micro hardness experimental summary. In this summary, the selected parameters are presented with their levels and the output response values such as micro hardness. Results of micro hardness values were clearly presented in the table. Higher values of micro hardness were recorded as 206.475 HV through the influence of reinforcement of 4%, stirring speed of 550 rpm, time of stirring 30 min and processing temperature 650 °C. In conclusion, the increase of process temperature can produce enhanced results.

Table 12 presents the response table for signal to noise ratios of micro hardness. This table properly showed the parameter influences and the levels of influence. Amongst the four process parameters, the process temperature parameter was extremely influenced and had the chief role in decision of micro hardness. The other three factors were influenced; compared to process temperature, they contributed a small amount. The second, third and fourth parameter influences are reinforcement %, stirring speed and time of stirring, respectively [43,44,45,46,47].

**Figure 16:**
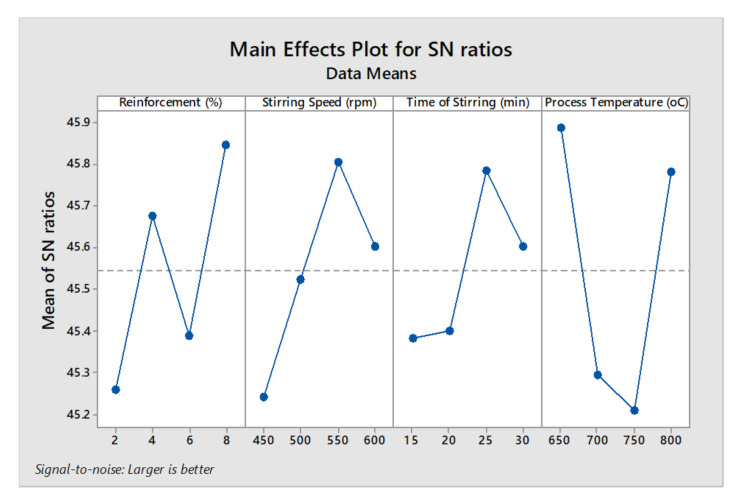
Main effect plot for SN ratios (micro hardness).

Figure 16 shows the main effect plot for SN ratios of micro hardness test; in this test, the four parameters partaking were discernibly presented. In reinforcement percentage involvement, the result values are in a zigzag manner such as increasing and decreasing of micro hardness values in a cyclic manner. Finally, the 8% of reinforcement offered the higher micro hardness values. In stirring speed parameter contribution, the increasing trends increase the micro hardness values. Further increases from 550 to 600 rpm led to decreased micro hardness values. The 550 rpm provided better micro hardness values. Considering the time of stirring parameters, the 25 min of stirring time offered excellent micro hardness values. Minimum level of temperature produces higher hardness values. Optimal parameters were obtained as 8% of reinforcement, 500 rpm of stirring speed, and 25 min of timing of stirring and 650 °C of processing temperature.

**Figure 17:**
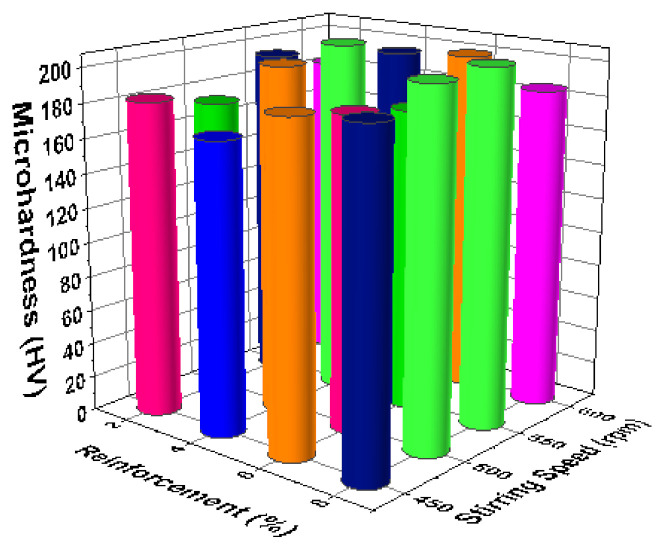
3D bar plot: reinforcement vs. stirring speed.

**Figure 18:**
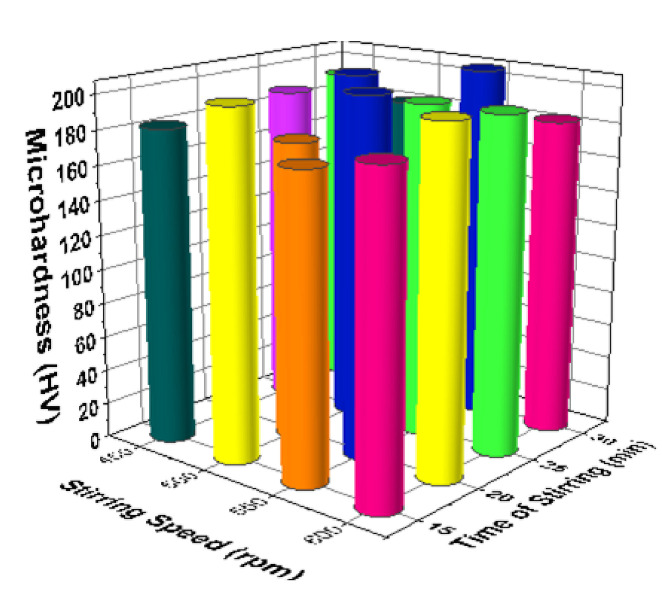
3D bar plot: stirring speed vs. time of stirring.

**Figure 19:**
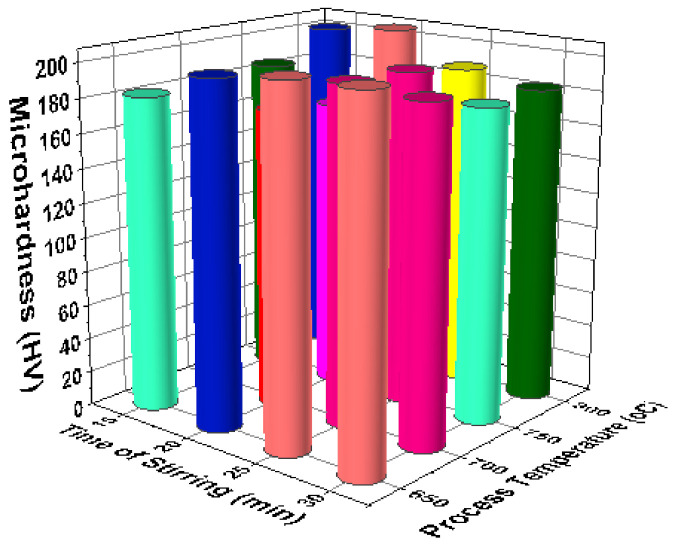
3D bar plot: time of stirring vs. process temperature.

**Figure 20:**
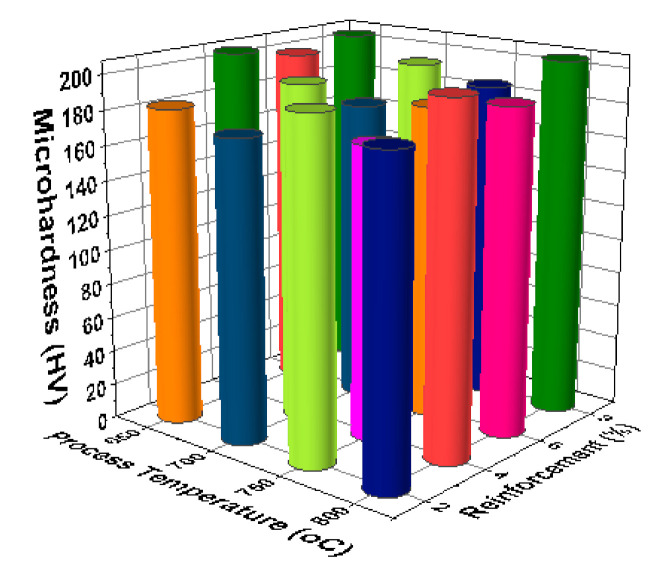
3D bar plot: process temperature vs. reinforcement %.

Figure 17 illustrates the 3D bar plot of the correlation between reinforcement and stirring speeds in the micro hardness analysis. Increase of stirring speed (550 rpm) and increase of reinforcement percentage (8%) offered excellent micro hardness values (around 200 HV). Figure 18 shows the correlation between stirring speed and time of stirring of the micro hardness analysis. The third level of stirring speed and minimum level of time of stirring encourage the better micro hardness values such as near to 200 HV. Figure 19 illustrates the relationship among the time of stirring and process temperature evidently. Influence of 25 min of time of stirring and 650 °C of processing temperature offered the higher levels of micro hardness values. Minimum micro hardness was recorded by using of 15 min of time of stirring and 750 °C of processing temperature. Figure 20 illustrates that for the connection between process temperature and reinforcement, the maximum micro hardness was recorded with influence of 750 °C of processing temperature and 8% of reinforcement.

**Figure 21:**
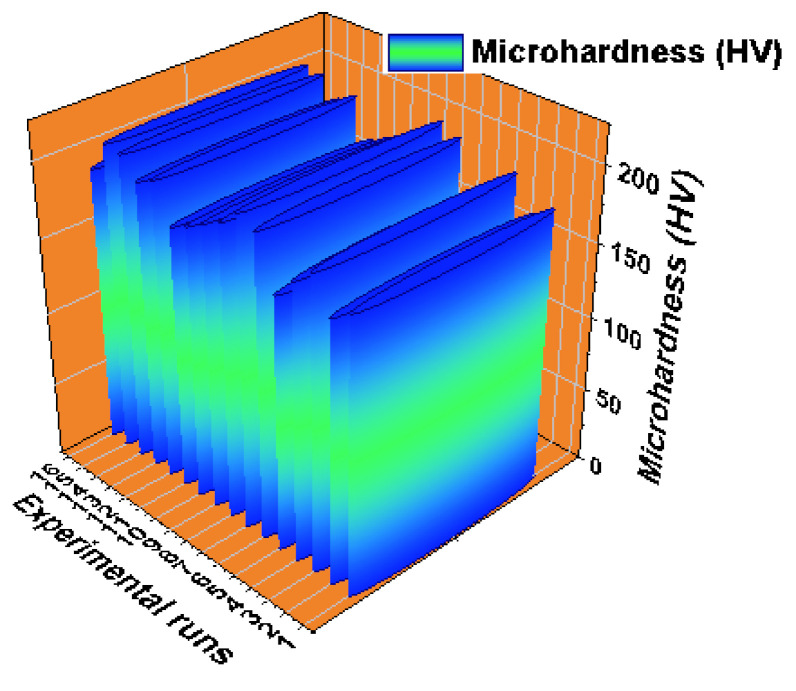
3D bar plot: experimental runs vs. micro hardness.

Figure 21 shows the 3D bar plot of experimental vs. micro hardness of the analysis. From all 16 experimental trials, each trial obviously exhibited the minimum and maximum micro hardness of the AZ61 hybrid composites. In most of the experimental trials, the micro hardness values slightly deviated from each other, For this inspection the maximum micro hardness was documented as 206.45 HV by the way of 4% of reinforcement, 550 rpm of stirring speed, 30 min of time of stirring and 650 °C process temperature. On other hand, the minimum micro hardness was registered as 168.54 HV with the effect of 4% of reinforcement, 450 rpm of stirring speed, 20 min of time of stirring and 750 °C process temperature. From this analysis, the several runs presented the exceptional micro hardness values. In all factors, the processing temperature extremely influenced the micro hardness.

## 5. Conclusions

The AZ61 magnesium alloy was produced by stir casting process with addition of born carbide + silicon carbide and the plate was made from the stir casting process successfully. The casted magnesium alloy was machined by wire cut EDM process. Surface roughness of the sliced out specimens in the casted magnesium alloy through wire cut EDM was checked using a surface roughness tester. The material removal rate, tensile strength and micro hardness tests were successfully carried out and the result of this investigation is as follows:

- The maximum material removal rate was obtained as 0.212 mm^3^/s influence of pulse on time of 115 µs, pulse off time of 50 µs. The contour plot illustrates the maximum MRR obtained as wire feed rate of 8 m/min and the pulse off time as 50 µs. The wire feed rate of 8 m/min and the current as 9 Amps offered good MRR. The minimum material removal rate was obtained as 0.069 mm^3^/s. Increasing of pulse on time slightly improved the MRR by the way of erosion. The highest wire feed rate increased the erosion rate, hence the MRR was achieved at the higher rate.
- The minimum and maximum values of surface roughness were obtained as 1.003942 and 3.203942 microns. From out of 16 specimens, the first specimen had minimum surface roughness value such as 1.0039 microns influenced by pulse on time of 105 µs, pulse off time of 40 µs, wire feed rate of 2 m/min and current of 3 Amps. The pulse off time parameter had the highest influencing factor among all four parameters. In surface roughness analysis, the optimal parameters were recorded as 105 µs of pulse on time, 40 µs of pulse off time, 8 m/min of wire feed rate and 6 Amps of current.
- From the tensile test, the maximum tensile strength was recorded as 245 MPa with influence of reinforcement of 2%, stirring speed of 600 rpm, time of stirring 30 min and processing temperature of 800 °C. Optimal parameters of the tensile test were obtained as 8% of reinforcement, 550 rpm of stirring speed, 30 min of stirring time period and 800 °C of processing temperature.
- In the micro hardness test, the higher values of micro hardness were recorded as 206.475 HV through influence of 4% of reinforcement, stirring speed of 550 rpm, time of stirring 30 min and processing temperature of 650 °C. Finally, the increase of process temperature in the stir casting process can be reflected in the enhanced results of surface roughness. Optimal parameters of the micro hardness test were obtained as 8% of reinforcement, 500 rpm of stirring speed, 25 min of stirring time and 650 °C of processing temperature.

## Figures and Tables

**Table 1:** Chemical composition of AZ61 magnesium alloy.

Material	% of Composition
Aluminum Al	6.510
Zinc Zn	1.092
Iron Fe	0.004
Nickel Ni	0.001
Manganese Mn	0.220
Silicon Si	0.040
Copper Cu	0.003
Magnesium Mg	92.13

**Table 2:** Mechanical properties of AZ61 magnesium alloy.

Properties	Metric MPa
Tensile strength	310
Yield strength	230
Compressive yield strength	130
Ultimate bearing strength	470
Bearing yield strength	285
Shear strength	140
Elongation at break	16%

**Table 3:** WCEDM process parameters.

Parameters	Values
Pulse on time (µs)	105, 110, 115 and 120
Pulse off time (µs)	40, 50, 60 and 70
Wire feed rate (m/min)	2, 4, 6 and 8
Current (Amps)	3, 6, 9 and 12

**Table 4:** Stir casting process parameters.

Parameters	Values
Reinforcement (%)	2, 4, 6 and 8
Stirring speed (rpm)	450, 500, 550 and 600
Time of stirring (min)	15, 20, 25 and 30
Process temperature (°C)	650, 700, 750 and 800

**Table 5:** Summary of WCEDM and material removal rate (MRR) results.

Exp. No	Pulse on Time (µs)	Pulse off Time (µs)	Wire Feed Rate (m/min)	Current (Amps)	Material Removal Rate (MRR) (mm^3^/s)
1	105	40	2	3	0.069
2	105	50	4	6	0.175
3	105	60	6	9	0.077
4	105	70	8	12	0.162
5	110	40	4	9	0.189
6	110	50	2	12	0.083
7	110	60	8	3	0.202
8	110	70	6	6	0.097
9	115	40	6	12	0.173
10	115	50	8	9	0.212
11	115	60	2	6	0.144
12	115	70	4	3	0.192
13	120	40	8	6	0.092
14	120	50	6	3	0.167
15	120	60	4	12	0.142
16	120	70	2	9	0.135

**Table 6:** Response table for signal to noise ratios (material removal rate).

Level	Pulse on Time (µs)	Pulse off Time (µs)	Wire Feed Rate (m/min)	Current (Amps)
1	−19.11	−18.41	−19.77	−16.75
2	−17.56	−16.44	−15.22	−18.24
3	−14.97	−17.49	−18.33	−16.90
4	−17.65	−16.95	−15.98	−17.41
Delta	4.14	1.97	4.54	1.49
Rank	2	3	1	4

**Table 7:** Outline of surface roughness results.

Surface Roughness (Ra), microns			
S. No	Test-1	Test-2	Average
1	0.66813	1.339755	1.003942
2	2.37413	2.001755	2.187942
3	2.94513	2.486755	2.715942
4	2.27413	2.155755	2.214942
5	2.30513	1.682755	1.993942
6	2.71513	3.190755	2.952942
7	2.67913	2.740755	2.709942
8	2.66313	2.435755	2.549442
9	1.53913	1.120755	1.329942
10	2.87013	3.128755	2.999442
11	3.13613	2.651755	2.893942
12	2.83613	2.707755	2.771942
13	1.31813	1.471755	1.394942
14	3.09813	3.148755	3.123442
15	3.06013	3.015755	3.037942
16	3.49613	2.911755	3.203942

**Table 8:** Response table for signal to noise ratios (surface roughness).

Level	Pulse on Time (µs)	Pulse off Time (µs)	Wire Feed Rate (m/min)	Current (Amps)
1	−5.605	−2.849	−7.196	−6.860
2	−8.047	−8.910	−7.826	−6.763
3	−7.526	−9.055	−7.294	−8.582
4	−8.137	−8.501	−7.000	−7.110
Delta	2.532	6.206	0.826	1.819
Rank	2	1	4	3

**Table 9:** Experimental summary of tensile strength.

Exp. Trials	Reinforcement (%)	Stirring Speed (rpm)	Time of Stirring (min)	Process Temperature (°C)	Tensile Strength (MPa)
1	2	450	15	650	208
2	2	500	20	700	214
3	2	550	25	750	236
4	2	600	30	800	245
5	4	450	20	750	217
6	4	500	15	800	228
7	4	550	30	650	237
8	4	600	25	700	208
9	6	450	25	800	219
10	6	500	30	750	220
11	6	550	15	700	238
12	6	600	20	650	244
13	8	450	30	700	236
14	8	500	25	650	230
15	8	550	20	800	241
16	8	600	15	750	218

**Table 10:** Response table for signal to noise ratios (tensile strength).

Level	Reinforcement(%)	Stirring Speed (rpm)	Time of Stirring (min)	ProcessTemperature (°C)
1	47.05	46.84	46.96	47.21
2	46.94	46.96	47.18	46.99
3	47.23	47.53	46.97	46.95
4	47.28	47.17	47.40	47.35
Delta	0.34	0.69	0.44	0.40
Rank	4	1	2	3

Larger is better.

**Table 11:** Experimental summary of micro hardness.

Exp. Trials	Reinforcement (%)	Stirring Speed (rpm)	Time of Stirring (min)	Process Temperature (°C)	Micro Hardness (HV)
1	2	450	15	650	182.40
2	2	500	20	700	173.65
3	2	550	25	750	194.27
4	2	600	30	800	183.12
5	4	450	20	750	168.54
6	4	500	15	800	201.27
7	4	550	30	650	206.45
8	4	600	25	700	195.17
9	6	450	25	800	188.67
10	6	500	30	750	181.42
11	6	550	15	700	175.38
12	6	600	20	650	199.24
13	8	450	30	700	192.71
14	8	500	25	650	204.73
15	8	550	20	800	206.18
16	8	600	15	750	185.41

**Table 12:** Response table for signal to noise ratios (micro hardness).

Level	Reinforcement (%)	Stirring Speed (rpm)	Time of Stirring (min)	ProcessTemperature (°C)
1	45.26	45.24	45.38	45.89
2	45.68	45.52	45.40	45.29
3	45.39	45.81	45.79	45.21
4	45.85	45.60	45.61	45.78
Delta	0.59	0.57	0.40	0.68
Rank	2	3	4	1

Larger is better.

## Data Availability

All the data is available within the manuscript.
